# Trend analysis of smoking-attributable hospitalizations in Thailand, 2007–2014

**DOI:** 10.18332/tid/98913

**Published:** 2018-11-05

**Authors:** Roengrudee Patanavanich, Wichai Aekplakorn, Paibul Suriyawongpaisal

**Affiliations:** 1 Faculty of Medicine, Ramathibodi Hospital, Mahidol University, Bangkok, Thailand

**Keywords:** smoking, Thailand, hospital admission, tobacco, attributable

## Abstract

**INTRODUCTION:**

Tobacco use is a major preventable risk factor for many noncommunicable diseases. Smoking-attributable mortality has been well described. However, the prevalence of smoking-attributable hospitalization (SAH) and associated costs have been less documented, especially in low- and middle-income countries. Our objective was to estimate the number of hospital admissions and expenditure attributable to tobacco use during 2007–2014 in Thailand.

**METHODS:**

Hospitalization data between 2007 and 2014 were used for the analysis. SAHs were derived by applying smoking-attributable fractions, based on Thailand’s estimates of smoking prevalence data and relative risks extracted from the published literature, to hospital admissions related to smoking according to the International Classification of Diseases version 10. Age-adjusted SAHs among adults age 35 and older were calculated. Joinpoint regression analysis was used to detect changes in trends among genders and geographical areas, based on annual per cent change (APC) and average annual per cent change (AAPC). Costs related to SAHs were also estimated.

**RESULTS:**

During 2007–2014, among adults age 35 years and older, smoking accounted for almost 3.6 million hospital admissions, with attributable hospital costs calculated at more than US$572 million annually, which represents 16.8% of the national hospital budget. While the age-adjusted rate of SAHs had been relatively stable (AAPC=1.12), the age-adjusted rate of SAHs due to cancers increased significantly for both sexes (AAPC=2.33). Cardiovascular diseases related to smoking increased significantly among men (AAPC=2.5), whereas, COPD, the most common smoking-related conditions decreased significantly during 2011–2014 (APC= -7.21). Furthermore, more provinces in the northeastern and the southern regions where smoking prevalence was higher than the national average have a significantly higher AAPC of SAH than other parts of the country.

**CONCLUSIONS:**

Smoking remains a significant health and economic burden in Thailand. Findings from this study pose compelling evidence for Thailand to advance tobacco control efforts to reduce the financial and social burden of diseases attributable to smoking.

## INTRODUCTION

Since the report of the U.S. Surgeon General in 1964 concluded that smoking was a cause of lung cancer, there has been growing awareness of the health risks of smoking worldwide. In 2014, the report of the U.S. Surgeon General revealed that cigarette smoking and exposure to secondhand smoke affect nearly every organ of the body; involving at least 15 types of cancer, various cardiovascular and respiratory diseases^1^. Recently, the World Health Organization (WHO) reports that one-fifth of the world population age 15 years and older are current smokers and smoking causes 7 million deaths each year^2,3^.

In Thailand, active antismoking movements began in 1986; the antismoking campaigns resulted in the legislation of new laws on tobacco control (the Tobacco Control Act of 1992 and the Non-Smokers Health Protection Act of 1992). Moreover, Thailand has ratified the WHO Framework Convention on Tobacco Control (WHO FCTC) since 2004^4^. Thereafter, several tobacco control policies were put in place, such as increasing taxes on tobacco products, ban on cigarette advertising, smoking-free zones, provincial antismoking programs, and health warning on tobacco products. Smoking prevalence among the population age 15 years and older in Thailand has fallen from 54.7% (male) and 6.1% (female) in 1976 to 37.7% and 1.7% in 2017^5,6^. Although knowledge has expanded dramatically on the health consequences of the diseases caused by tobacco use and involuntary exposure to tobacco smoke, studies on health outcomes related to smoking in Thailand are relatively rare.

Smoking-attributable mortality (SAM), a health impact indicator for tobacco control has been well described. Many countries have used smoking-attributable fraction (SAF) to estimate SAM for monitoring and evaluation of their tobacco control policies^7^. Some of these countries have extended the use of SAF to hospitalization data to estimate smoking-attributable hospitalization (SAH)^7-9^. However, SAH has been less documented, particularly in low- and middle-income countries (LMICs). To assist antismoking efforts aimed at reducing smoking prevalence in Thailand, and to help reduce the health consequences attributable to tobacco use, information on the effects of smoking on morbidity is needed. Our objective was to estimate the number of hospital admissions and costs attributable to tobacco use between 2007 and 2014 in Thailand.

## METHODS

### Data sources

To calculate the proportion of SAHs attributable to tobacco use, data on the prevalence of smoking, the relative risk of smokers developing a certain disease or condition, and the number of hospital admissions for such disease or condition is required^8^.

Smoking prevalence data for the Thai population were obtained from the 2011 National Cigarette Smoking and Alcohol Drinking Behavior Survey, collected by the National Statistical Office^10^.

There is no comprehensive cohort study in Thailand that collects data on smoking behavior and health-related outcomes to obtain the relative risk. Therefore, a list of smoking-related conditions and their relative risks included in this study were based on the 2014 Health Consequences of Smoking–50 Years of Progress: A Report from the Surgeon General1.The report provides the widely acceptable rubric of smoking-related disease categories and the associated relative risk values that are based on the first 6 years of follow-up of the Cancer Prevention Study II during 1982–1988 (CPS-II), one of the largest U.S. cohort studies that collects smoking information^1^.

Inpatient de-identified discharge data between 2007 and 2014 from three major health insurance schemes (universal coverage, social security, and civil servant schemes – accounted for nearly 99% of the Thai population) were obtained from the National Health Security Office, the Social Security Office and the Health Insurance System Research Office^11^. The information presented in this study was based on any diagnosis for each hospital admission (the principal diagnosis and other up to 12 sub-diagnoses). The discharge data also provided information on total hospital charge for each admission.

Population estimates were based on 2007–2014 Thailand population statistics from Thailand’s Official Statistics Registration Systems, Department of Provincial Administration and the world standard population from the WHO^12,13^.

### Calculation of smoking-attributable hospitalizations

To quantify the contribution of smoking as a risk factor for hospital admissions, the SAF for such conditions must be identified. SAF is a function of the prevalence of smoking and the relative risk function of each smoking-related condition, and its calculation enables the estimation of the proportion of cases of a disease that may be attributed to the use of tobacco. The SAFs for chronic diseases were calculated by Levin’s formula of population attributable fraction^14,15^:

SAF = Σ_i_ (P_i_ (RR_i_ - 1)) / Σ_i_ (1 + P_i_ (RR_i_ - 1))

where *P_i_* = prevalence of smoking in group *i*, with *RR_i_* = relative risk in exposed group i compared with the unexposed group, and *Σ_i_* is a summation over each *i* corresponding to three exposure groups (non-smoker, former smoker, current smoker).

The SAFs were calculated across the three categories, non-smoker, current smoker, and former smoker, for each sex, then combined to estimate the overall SAF for both sexes for each disease.

To calculate the proportion of SAHs, the SAF was applied to the total number of hospital admissions for each condition. Because the relative risks used in this study were based on adults age 35 years and older, the calculation of SAHs in the present study was based on the number of hospitalizations among adults age 35 years and older.

In addition, any diagnosis was used in the analysis of the SAHs to reduce underestimation of the number of hospitalizations due to smoking when only principal diagnosis is considered^16^. Nevertheless, one admission may contain multiple smoking-related diagnoses; therefore, to prevent double counting, the diagnosis with the highest SAF was used for the calculation of the overall SAHs and each disease category. For example, if a patient was admitted to a hospital with COPD, lung cancer, and ischemic heart disease, this patient would be counted in each disease category (tobacco-related respiratory disease, tobacco-related cancer, and tobacco-related CVD), but would be counted only once in the overall SAHs.

### Calculation of total hospital costs associated with smoking-attributable hospitalizations

Costs related to each hospital admission were calculated by multiplying a ratio of cost to charge (obtained from the study of standard cost lists for health technology assessment in Thailand) to the total hospital charge for each admission^17^. Then, the costs of SAHs were estimated according to the SAF of each condition.

### Trend analysis of smoking-attributable hospitalizations

The direct age-standardization method was applied to calculate the age-adjusted rate of SAHs. Time trends in the annual rate of the age-adjusted rate of SAHs were examined using joinpoint regression analysis to detect changes in annual per cent change (APC) and average annual per cent change (AAPC). The trends were generated for both sexes, men and women.

AAPCs calculated by joinpoint regression analysis uses the annual per cent changes from segmented analysis to summarize and compare trends for a specific time period. The advantage of AAPCs is that it takes into account the trend transitions; whereas, the conventional annual per cent change does not and can lead to inaccurate conclusions^18^.

In addition, the AAPCs during 2007–2014 for each province and geographical region were calculated and compared with those at country level. We used Joinpoint version 4.6.0.0^19^.

## RESULTS

**Figure 1:**
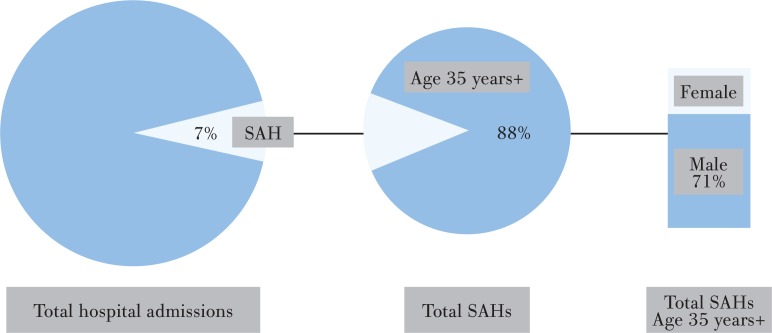
Proportion of SAHs in Thailand, 2007–2014

During 2007–2014, for all age groups, SAHs accounted for 4057791 hospital admissions, representing 7.3% of the total number of hospital admissions in Thailand. SAHs among adults age 35 years and older accounted for 88.1% or 3574728 admissions (71% male and 29% female; Figure 1).

SAHs among adults age 35 years and older were attributed to 3 major disease categories: tobacco-related cancers (8.8%), tobacco-related cardiovascular diseases (46.6%), and tobacco-related respiratory diseases (41.8%). The most common conditions of smoking-related hospitalizations were COPD (J44) with 1240128 admissions (34.7%), ischemic heart diseases (I20-I25) with 734453 admissions (20.5%), cerebrovascular diseases (I60-69) with 597100 admissions (16.7%), and other circulatory diseases (I00, I26-I51) with 589500 admissions (16.5%). Lung cancer (C33-C34) was the leading tobacco-related cancer with 249640 admissions (7.0%), followed by oropharyngeal cancer (C00-C14) with 112858 admissions (3.2%). The SAHs for each smoking-related condition are presented in Table 1.

**Table 1:** Tobacco-related conditions, corresponding ICD-10 codes, SAHs and SAFs among adults age 35 years and older in Thailand, 2007–2014

*Condition*	*Group of condition*	*ICD 10*	*SAH 2007–2014*			*SAF*		

			*Total*	*Male*	*Female*	*Total*	*Male*	*Female*
Oropharyngeal	**Tobacco-related cancers**	C00-C14	112858	89642	19776	0.734	0.826	0.437
Esophageal		C15	36401	34292	4374	0.679	0.760	0.515
Stomach		C16	18671	12848	6142	0.285	0.337	0.224
Pancreatic		C25	9902	6445	3214	0.297	0.373	0.200
Laryngeal		C32	26805	26720	1549	0.810	0.874	0.613
Lung		C33-C34	249640	169373	69147	0.868	0.918	0.672
Cervical		C53	28	0	28	0.002	0.000	0.002
Kidney		C64-C65	795	618	197	0.380	0.470	0.253
Urinary bladder		C6	28837	27724	4030	0.449	0.544	0.304
AML		C92	7394	4281	2985	0.257	0.304	0.203
**Tobacco-related cancers**	**Tobacco-related cardiovascular diseases**		**491331**	**371944**	**111443**	**0.669**	**0.758**	**0.458**
Ischemic heart		I20-I25	734453	476637	238887	0.389	0.475	0.271
Other heart		I00, I26-I51	589500	308659	266078	0.237	0.275	0.195
Cerebrovascular		I60-I69	597100	422265	150043	0.387	0.496	0.217
Atherosclerosis		I70	4263	2779	1359	0.324	0.407	0.215
Aortic aneurysm		I71	28826	21301	7094	0.631	0.727	0.434
Other arterial		I72-I78	15988	10758	5195	0.255	0.316	0.180
**Tobacco-related cardiovascular diseases**	**Tobacco-related respiratory diseases**		**1970130**	**1242400**	**668655**	**0.326**	**0.408**	**0.224**
Influenza		J10-J11	16704	7778	8543	0.255	0.283	0.225
Pneumonia		J12-J18	391105	234185	158694	0.255	0.283	0.225
Bronchitis emphysema		J40-J43	19581	11582	7559	0.869	0.908	0.773
COPD		J44	1240128	1021763	238876	0.777	0.841	0.627
TB		A16-A19	99062	72674	29595	0.255	0.283	0.225
**Tobacco-related respiratory diseases**			**1766580**	**1347981**	**443268**	**0.490**	**0.576**	**0.350**
All smoking-related conditions**			**3574728**	**2538312**	**1036416**	**0.437**	**0.554**	**0.289**

*Smoking prevalence based on the 2011 National Cigarette Smoking and Alcohol Drinking Behavior Survey.

** One admission may contain multiple smoking-related diagnoses (the diagnosis with the highest SAF was used for calculation).

### The trends of age-adjusted rate of smoking-attributable hospitalizations

The trend of age-adjusted rate of the overall SAHs increased over the period 2007 to 2014 (AAPC=1.12), but insignificantly.

For tobacco-related cancers, the trend of age-adjusted rate increased significantly between 2007 and 2014 (AAPC=2.33).

For tobacco-related cardiovascular diseases, although their SAFs were not as high as tobacco-related cancers and tobacco-related respiratory diseases, the number of SAHs was dominant. The trend of age-adjusted rate among men increased significantly over time (AAPC=2.5); whereas the trend among women increased insignificantly.

**Figure 2:**
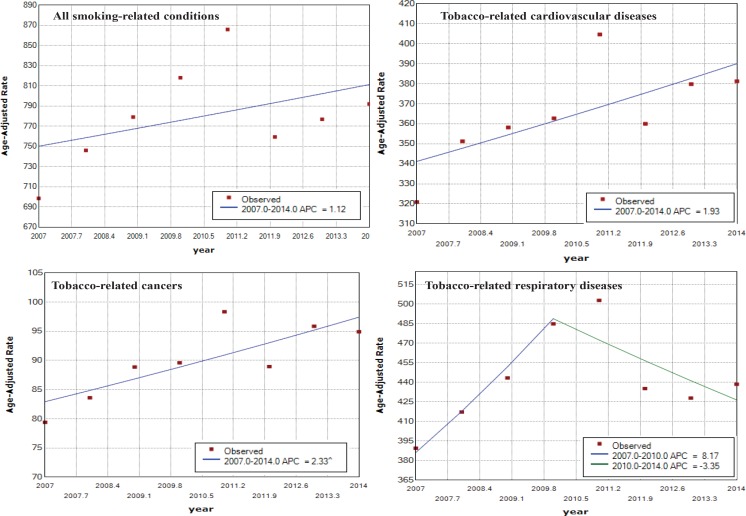
Trends in age-adjusted rates of SAHs (per 100000) in Thailand by disease groups, 2007–2014

For tobacco-related respiratory diseases, the overall trend of age-adjusted rate increased from 2007 to 2014 (AAPC=1.4), but the trend decreased significantly over the period 2010 to 2014 (APC=-3.35). These results are shown in Table 2 and Figure 2.

**Table 2:** Trends in age-adjusted rates of SAHs (per 100000) in Thailand by disease groups, 2007–2014

*Groups*	*Joinpoint regression*				
	*Trend 1 Period*	*APC*	*Trend 2 Period*	*APC*	*AAPC 2007–2014*
		**All smoking-related conditions**			
Both sexes	2007–2014	1.12			1.12
Men	2007–2014	1.17			1.17
Women	2007–2014	0.85			0.85
		**Tobacco-related cancers**			
Both sexes	2007–2014	2.33*			2.33*
Men	2007–2014	2.30*			2.30*
Women	2007–2014	2.29*			2.29*
		**Tobacco-related cardiovascular diseases**			
Both sexes	2007–2014	1.93			1.9
Men	2007–2014	2.50*			2.5*
Women	2007–2014	0.74			0.70
		**Tobacco-related respiratory diseases**			
Both sexes	2007–2010	8.17	2010–2014	-3.35*	1.40
Men	2007–2010	7.23	2010–2014	-3.27	1.10
Women	2007–2010	10.25	2010–2014	-3.67	2.10

APC: annual per cent change. AAPC: average annual per cent change.

* significantly different from zero at alpha=0.05 level.

### The trend of age-adjusted rate of selected smoking-attributable hospitalizations

**Figure 3:**
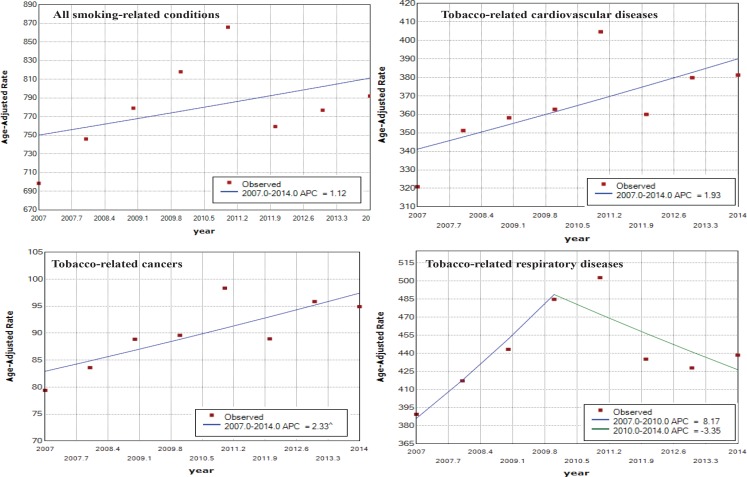
Trends in age-adjusted rates of SAHs (per 100000) in Thailand by selected conditions, 2007–2014

We calculated the age-adjusted rates of selected SAHs, where the SAFs were higher than 70%, to examine their trends over the period 2007 to 2014; these results are shown in Table 3 and Figure 3. The conditions with corresponding SAF were: COPD (SAF=0.78), bronchitis emphysema (SAF=0.87), oropharyngeal cancer (SAF=0.73), laryngeal cancer (SAF=0.81) and lung cancer (SAF=0.87).

**Table 3:** Trends in age-adjusted rates of SAHs (per 100000) in Thailand by selected conditions, 2007-2014

*Groups*	*Joinpoint regression*				
	*Trend 1 Period*	*APC*	*Trend 2 Period*	*APC*	*AAPC 2007–2014*
		**COPD**			
Both sexes	2007–2011	3.33*	2011–2014	-7.21*	-1.30
Men	2007–2011	3.02*	2011–2014	-6.72*	-1.30
Women	2007–2011	4.38*	2011–2014	-8.99*	-1.60
		**Bronchitis emphysema**			
Both sexes	2007–2014	-4.93*			-4.93*
Men	2007–2011	0.94	2011–2014	-7.81*	-2.90*
Women	2007–2014	-8.50*			-8.50*
		**Lung cancer**			
Both sexes	2007–2014	1.95			1.95
Men	2007–2014	1.49			1.49
Women	2007–2014	3.08*			3.08*
		**Laryngeal cancer**			
Both sexes	2007–2010	3.99	2010–2014	-0.79	1.20
Men	2007–2010	5.04*	2010–2014	1.05	1.50
Women	2007–2014	-4.22*			-4.22*
		**Oropharyngeal cancer**			
Both sexes	2007–2014	2.44*			2.44*
Men	2007–2014	3.06*			3.06*
Women	2007–2014	-0.35			-0.35

APC: annual per cent change. AAPC: average annual per cent change.

* significantly different from zero at alpha=0.05 level.

During 2007–2014, hospital admissions due to COPD attributable to smoking were 1240128 admissions (29.3% of all SAHs). Interestingly, the trend of age-adjusted rate of COPD decreased during the period 2007 to 2014 (AAPC= -1.3), with a significant decrease between 2011 and 2014 (APC= -7.21). The downward trend was seen in both men and women.

The trend for bronchitis emphysema attributable to smoking was in the same direction as COPD; significantly decreased over the period 2007 to 2014 (AAPC= -4.93).

For the most common tobacco-related cancers, the trend of age-adjusted rate of lung cancer among women increased significantly (AAPC=3.08); however, the trend of age-adjusted rate of laryngeal cancer decreased significantly (AAPC= -4.22). The trend of age-adjusted rate of oropharyngeal cancer increased significantly (AAPC=2.44).

### The average annual per cent change during 2007–2014 at the provincial level and its relationship with smoking prevalence

**Figure 4:**
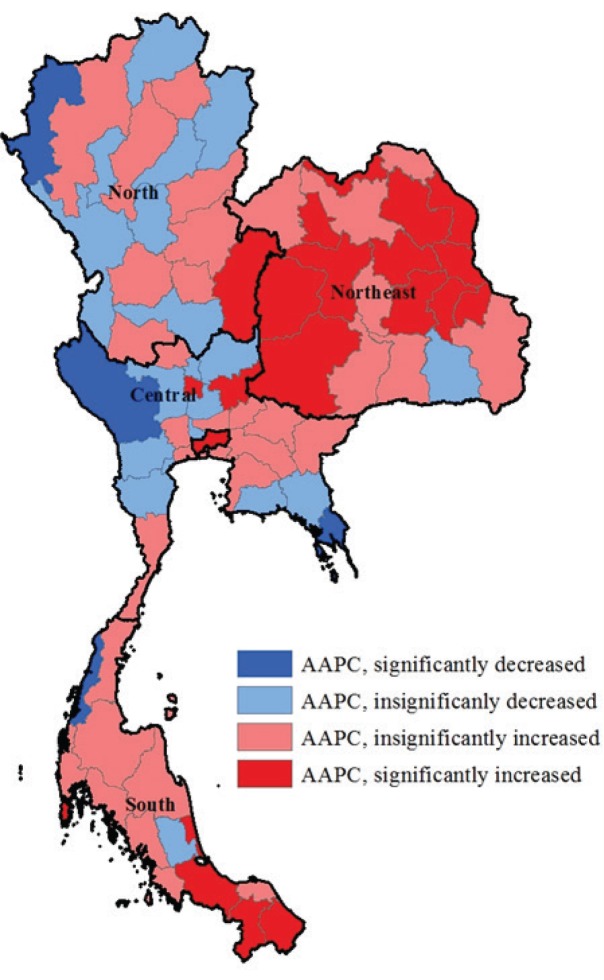
AAPCs of the overall SAHs by province, Thailand, 2007–2014

During 2007–2014, of all 76 provinces, there were 23 provinces with a decrease in AAPC of the overall SAHs. Among these 23 provinces, the trends of age-adjusted rate of SAHs in 4 provinces decreased significantly (Kanchanaburi, Mae Hong Son, Ratchaburi, Trat). In contrast, the AAPCs increased significantly in 20 provinces; most of the provinces were in the southern and northeastern regions where the smoking rates were generally higher than in other parts of the country. The northeastern region had the highest number and proportion of provinces with a significant increase in AAPCs (63.2%) followed by the southern (28.6%), whereas for the central and northern regions values were far lower than the national average. The AAPC of the age-adjusted rate of the overall SAHs by province is shown in Figure 4. The prevalence of smoking by geographical region and the AAPCs are presented in Table 4.

**Table 4:** Smoking prevalence by geographical region and AAPC of SAH, 2007–2014

*Geographical region*	*Average smoking prevalence*	*Per cent of provinces with an increase in AAPC of SAH*	*Per cent of provinces with a significant increase in AAPC of SAH*
**Thailand**	21.1	43.4	26.3
Central	19.0	20.0	8.0
North	21.0	23.5	5.9
North–East	23.1	84.2	63.2
South	25.9	50.0	28.6

*Prevalence data from the National Cigarette Smoking and Alcohol Drinking Behavior Survey.

### Hospital costs associated with treating smoking-attributable hospitalizations among adults age 35 years and older

During 2007–2014, hospital costs associated with treating SAHs were US$4578 million (Exchange rate: US$1=33.2 THB) or an average of $572 million per year. Interestingly, treatment of SAHs accounted for $1 of every $6 spent on all inpatient care. Moreover, the proportion of costs for SAHs increased from 16.3% in 2007 to 18.3% in 2014. Hospital costs for each smoking-related condition are shown in Table 5.

**Table 5:** Total hospital costs associated with SAH among adults age 35 years and older in Thailand, 2007–2014

*Group of condition Total costs*	*(million US$)*	*Average costs per year (million US$)*	*Average costs per SAH (US$)*
Tobacco-obacco-related cancers	664	83	1351
Tobacco-related cardiovascular diseases	2738	342	1390
Tobacco-related respiratory diseases	1828	228	1034
All smoking-related conditions*	4578	572	1082

Exchange rate: US$1=33.2 THB.

* One admission may contain multiple smoking-related

Cardiovascular diseases, attributable to smoking, cost $342 million per year. In addition, average cost per SAH due to cardiovascular diseases was the most expensive ($1390 per admission); whereas the average costs per SAH due to cancers and respiratory diseases were $1351 and $1034, respectively. The proportion of costs for cardiovascular SAHs increased from 56.5% of costs for all SAHs in 2007 to 62.9% in 2014. However, the proportion of costs for respiratory SAHs decreased from 15.9% in 2007 to 13.6% in 2014. The proportion of costs for tobacco-related cancers was relatively stable over the same period.

## DISCUSSION

Despite vigorous anti-smoking movements initiated by government and non-government agencies, smoking-attributable morbidities and their expenditures show no detectable decline.

In fact, studies in many countries show that disease burden attributable to smoking generally increases despite the reduction in smoking prevalence^1,20,21^. Passive smoking could be one explanation. In the present study it was found that the male-to-female overall SAH ratio was inconsistent with the current male-to-female smoking prevalence ratio (2.4 vs 22.2). This finding indicates that the high morbidity among women, even though smoking prevalence is relatively low, may be due to passive smoking, however, this issue needs further investigation. Another explanation could be the latency between smoking exposure and disease onset^22-25^. In addition, a risk reduction of smoking-related diseases was observed after several years of smoking cessation. For instance, a study indicated that the excess risk of developing COPD, lung cancer, and ischemic heart disease become half following quitting smoking with varying duration: 13.3, 9.9, and 4.4 years, respectively^24, 26^. Furthermore, a well-established model indicates that for the substantial health hazards of tobacco use in developed countries there is a three to four decade lag between the peak in the prevalence of smoking and the subsequent peak in smoking-attributable mortality^27,28^. However, the lag time between the smoking prevalence and the smoking-related mortality and morbidity in low- and middle-income countries has not been established.

The timeframe of this study is too short to determine at what stage Thailand’s smoking epidemic curve is currently at; nevertheless, with continuous monitoring, this will become clearer over several years. For example, the significant reduction in the average annual per cent change of COPD found in this study in the last four years could be a result of the huge decline in smoking prevalence in Thailand more than decades ago. Average annual per cent changes during the study period might also be a result of significant increased access to healthcare subsequent to the commencement of the universal health coverage in 2002^29^. The increasing trend in age-specific rate of SAHs emphasizes the need for more intensive anti-smoking interventions over an extended time span. It also indicates a need for strengthening primary care to minimize the needs for hospitalization among patients with smoking-related diseases^30^.

The present study also demonstrates that Thailand spends about $572 million annually on treating patients admitted to hospitals because of smoking and the costs have tended to increase over time (16.3% of the total hospital cost in 2007 to 18.3% in 2014). Furthermore, there are other costs attributable to smoking, such as direct medical costs in outpatient units and other indirect costs. A study in 2009 estimated overall economic loss in Thailand due to smoking was $2255 million annually; whereas the excise department reported that the annual tobacco tax collection was only $1323 million^31,32^. This seemingly net economic loss from cigarette smoking helps to justify population-based interventions targeting reduction in tobacco use. In effect, recent evidence from a systematic review substantiates the cost-effectiveness of the interventions in low- and middle-income countries with potential to generate economic gains that can be reinvested to improve health and/or other sectors^33^.

Globally socioeconomic inequalities in all forms of tobacco use are well established^34^ and our findings on the distributions of SAHs among the genders (Table 2) and geographical regions (Table 4) conform to this inequality patterns. The average annual per cent change of SAHs in the northeast and the south seems to be higher than in other regions. Concurrently, smoking prevalence rates of the northeast and the south are generally higher than in other regions. The geographic variation of SAHs in Thailand can be caused by several factors. Different patterns of cigarette smoking could be one leading cause, as more than 60% of people in the northeastern region, a higher rate than in other regions, prefer hand rolled cigarettes that are much cheaper than regular cigarettes due to lower tax rates^10^. Other factors are lower socioeconomic characteristics such as income and education compared to others. The northeastern region also has the lowest physician-to-population ratio and poor access to smoking cessation services.

There are limitations of the study. This study is based on hospitalization data only and so could underestimate effects of tobacco-related morbidity, especially those who did not receive care in a hospital or were treated in outpatient departments. Also, misdiagnosis or wrongly assigned a diagnosis-related group (DRG) coding is possible^35^. Furthermore, variations in access to care by region may exist. Moreover, accuracy and completeness of the fill-in data may be different by region. Lastly, this study did not include the effects of secondhand smoke.

The present study has strengths by including three national data sources that cover almost all hospitalizations of the Thai population and by showing a time trend. In addition, the advantage of using hospital discharge data is that they are a readily available to the public, periodically updated, and are a nationally representative data set^36^. Hence long-term monitoring of health impacts of tobacco use is enabled using hospital discharge data. Finally, our study complements the conventional surveys on smoking prevalence.

## CONCLUSIONS

Smoking remains a significant health and economic burden in Thailand. Although, Thailand has made good progress on implementing many smoking control interventions recommended by the FCTC, the current sluggish decline in smoking prevalence indicates that further improvements are needed to increase effectiveness of the programs and reduce diseases caused by smoking. Findings from this study pose a compelling reason for Thailand to advance tobacco control efforts to reduce the financial and social burden of disease caused by smoking. Emphasis on more effective implementation of population-based interventions could prevent youth smokers and motivate attempts to quit among current smokers. In addition, strengthening primary care could prevent unnecessary hospitalization of patients with smoking-related diseases and reduce healthcare costs.

## CONFLICTS OF INTEREST

Authors have completed and submitted the ICMJE Form for Disclosure of Potential Conflicts of Interest and none was reported.
